# Ecological interactions shape the evolution of flower color in communities across a temperate biodiversity hotspot

**DOI:** 10.1002/evl3.225

**Published:** 2021-04-28

**Authors:** Alexander Skeels, Russell Dinnage, Iliana Medina, Marcel Cardillo

**Affiliations:** ^1^ Division of Ecology and Evolution, Research School of Biology Australian National University Canberra ACT 0200 Australia; ^2^ Landscape Ecology, Institute of Terrestrial Ecosystems, Department of Environmental Systems Science ETH Zürich Zürich CH‐8092 Switzerland; ^3^ Unit of Land Change Science, Swiss Federal Research Institute for Forest Snow and Landscape WSL Birmensdorf CH‐8903 Switzerland; ^4^ Institute for Applied Ecology University of Canberra Canberra ACT 2617 Australia; ^5^ School of BioSciences University of Melbourne Melbourne VIC 3010 Australia

**Keywords:** Competition, floral traits, macroevolution, pollination ecology, Proteaceae, reproductive interference

## Abstract

Processes driving the divergence of floral traits may be integral to the extraordinary richness of flowering plants and the assembly of diverse plant communities. Several models of pollinator‐mediated floral evolution have been proposed; floral divergence may (i) be directly involved in driving speciation or may occur after speciation driven by (ii) drift or local adaptation in allopatry or (iii) negative interactions between species in sympatry. Here, we generate predictions for patterns of trait divergence and community assembly expected under these three models, and test these predictions in *Hakea* (Proteaceae), a diverse genus in the Southwest Australian biodiversity hotspot. We quantified functional richness for two key floral traits (pistil length and flower color), as well as phylogenetic distances between species, across ecological communities, and compared these to patterns generated from null models of community assembly. We also estimated the statistical relationship between rates of trait evolution and lineage diversification across the phylogeny. Patterns of community assembly suggest that flower color, but not floral phenology or morphology, or phylogenetic relatedness, is more divergent in communities than expected. Rates of lineage diversification and flower color evolution were negatively correlated across the phylogeny and rates of flower colour evolution were positively related to branching times. These results support a role for diversity‐dependent species interactions driving floral divergence during the Hakea radiation, contributing to the development of the extraordinary species richness of southwest Australia.

**Impact Summary**

Plants have a stunningly diverse array of floral shapes, sizes, and colors, and it has long been suspected that this floral diversity explains why flowering plants are so species‐rich today. Regulation of pollen transfer between flowers is key to the formation of reproductive barriers, the heart of the speciation process, so flowers may be under strong selection to diverge during speciation. Alternatively, changes in floral traits may happen after speciation is complete, as species adapt to new environments and pollinators. A third option is that species with similar flowers may compete for pollinators when they occur together in the same communities, which may accelerate changes in flowers to gain new pollinators or a greater share of available pollinators. To distinguish these scenarios, we need to look at the floral traits of species that co‐occur within ecological communities and reconstruct the evolutionary history of these traits. As a case study, we examine the Australian genus *Hakea*, in one of the most diverse temperate ecosystems in the world, Southwest Australia. Using digital photographs, we show that species that coexist in communities have very different flower colors, more so than we expect by chance alone, which is a possible signature of competition. Although the genus is roughly 35 million years old, much of this diversity in flower color evolved after the Mid‐Miocene (15 million years ago). Potential for competitive interactions between *Hakea* species should be density dependent—increasing as the number of co‐occurring species increases—so greater trait diversification toward the present is consistent with an increasing influence of competition. Together, our results support the role of competition being an important driver of flower color evolution in *Hakea*, which helps us better understand how many close relatives coexist in high numbers, shaping a biodiversity hotspot.

How do numerous plant species coexist in diverse ecological communities? Closely related species are most likely to compete for resources (e.g., space, light, nutrients, water) due to shared ecological characters inherited from a recent common ancestor (Darwin 1859; Elton 1946). Closely related species may also share similar reproductive morphology, phenology, pollinator signals, and pollinator animal vectors. In some cases, this may lead to strong negative biotic interactions such as competition for pollinators (Levin and Anderson 1970) or reproductive interference, where species bear a cost from heterospecific pollen flow (Moreira‐Hernández and Muchhala 2019). In other cases, similar pollination strategies may be advantageous if shared floral resources increase pollinator abundance and visitation rates (Junker et al. 2015). One of the major challenges to coexistence in close relatives is managing trade‐offs between potential benefits of floral similarity and the pitfalls of negative interactions. To understand the role of pollinators in driving plant diversity, we need to understand how plant‐pollinator systems evolve over macroevolutionary timescales, and how this influences species distributions and floral traits in present‐day communities (Sauquet and Magallón 2018).

To reduce negative interactions, species may diverge along distinct pollination niche axes, including flowering phenology, floral morphology, or pollinator signaling, each operating as a prezygotic barrier to reproductive interference by minimizing synchronous use of the same pollinator vectors (Schiestl and Schlüter 2009; Baack et al. 2015). Flowering at different times reduces the potential for pollen sharing between individuals of different species. However, flowering phenology in seasonal environments is often constrained by the availability of resources including water, pollinators, and the timing of other life history events such as fruiting (Forrest and Miller‐Rushing 2010), which may promote local synchrony in flowering time. If species flower in synchrony, there may be pressure to diverge along at least one of two other pollination niche axes (Armbruster et al. 1994; Eaton et al. 2012). Divergence in morphological traits such as flower size alters the mechanics of pollination and determines which animal vectors can receive and deliver pollen, whereas floral signaling traits, including scent or color, serve to attract pollinators and can be generalized or specialized to service different animal vectors (Grant 1994). Evidence from floral trait patterns in ecological communities typically supports the role of divergence between close relatives along at least one of these three niche axes (e.g., Aizen and Vázquez 2006; Eaton et al. 2012; Muchhala et al. 2014; Weber et al. 2018). However, how the variation in pollination traits has evolved over deep‐time and is related to diversification remains an open question (Van der Niet and Johnson 2012; Sauquet and Magallón 2018; Vamosi et al. 2018; Hernández and Wiens 2020).

Alternative mechanisms (models 1–3; Fig. 1) of floral trait evolution are amenable to testing using comparative data because they predict distinct patterns in reconstructions of their macroevolutionary history and community patterns (Armbruster and Muchhala 2009; Roncal et al. 2012; Weber et al. 2018).

(1) *Speciational model*. If floral traits are involved in driving or reinforcing speciation (Fig. 1), rapid diversification will be coupled with rapid floral trait divergence, and so we expect to see a positive relationship between the rates of lineage diversification and floral trait disparification across branches of the phylogeny (rates correlation; Fig. 1). Because speciation is associated with changes in floral traits, trait differences should not be well predicted by phylogenetic relatedness, or in other words, traits should show low phylogenetic signal (phylogenetic signal; Fig. 1). Communities on the other hand would be expected to contain close relatives (and show phylogenetic clustering) because the speciation process places florally divergent sister species in sympatry (community phylogenetics; Fig. 1). Negative interactions are key to driving reproductive isolation under the speciational model; however, the macroevolutionary predictions differ to the (post‐speciational) negative interactions model below in being positively diversity dependent—as lineages increase in number, so too does the effect of competition and this promotes further ecological opportunity for speciation (Rundle and Nosil 2005; Chira et al. 2020).
(2) *Allopatric divergence model*. Alternatively, if floral traits diverge gradually in allopatric populations due to random drift in phenotypes or adaptation to different environments and pollinator communities, we do not expect a relationship between lineage diversification and floral trait diversification rates, as we expect trait differences to be largely a function of time since divergence, and therefore support a Brownian motion (BM) model of trait evolution (or one of its variants), with phylogenetic signal consistent with this pattern. Under this model, recently diverged sister species will be the most similar in floral traits and communities of florally divergent species, expected if competition is shaping community assembly, should be assembled from more phylogenetically distant lineages.
(3) *Negative interactions model*. Finally, if floral traits diverge as a result of selection to minimize competition or reproductive interference among sympatric lineages (Pfennig and Pfennig 2009), then we expect floral trait diversification to be associated with the potential for competition among species. As the number of lineages increases in a region, so do the number of potential interspecific interactions. This has the twofold effect of decreasing the rate of diversification, due to reduced opportunity for ecological speciation (Schluter 2000; Phillimore and Price 2008), and increasing the rate of floral trait divergence, to minimize negative interactions between interacting species. Therefore, this model is negatively diversity dependent and the rate of lineage diversification should be negatively correlated with the rate of floral trait evolution. This model makes no predictions about the phylogenetic structure of communities because communities of florally divergent species may be assembled from closely related or distantly related species.

**Figure 1:**
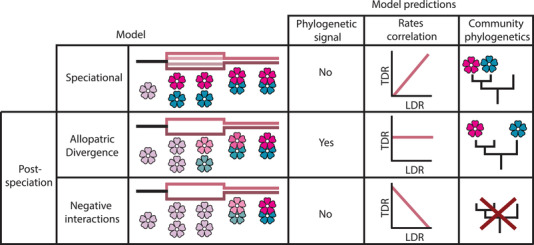
Predictions for macroevolutionary patterns in floral trait divergence and lineage diversification under three alternative models. Model illustrations show the branching of a lineage into two new species in allopatry (or also in sympatry as shown in the speciational model) before returning to sympatry. The speciational model predicts the timing of phenotypic change to be associated with the divergence of the lineages, as illustrated by a change in flower color at the speciation event. The allopatric divergence model predicts gradual change over time, as illustrated by a gradual gradient in flower color change along the branches. The negative interactions model predicts changes to be driven by interspecific interactions in sympatry, as illustrated by a shift in flower color at the point in which the species return to sympatry. These three models have different predictions for the presence of phylogenetic signal in traits values, for the correlation between rates of trait disparification (TDR) and lineage diversification rate (LDR), and the phylogenetic structure of ecological communities (explained in detail in the main text).

In this study, we aim to explore how the macroevolutionary history of floral trait diversification has contributed to contemporary structure and diversity of ecological communities. Specifically, we want to know (1) do ecological communities exhibit nonrandom patterns of floral trait diversity (similarity or divergence) along different pollination‐niche axes? (2) Do phylogenetic patterns of floral trait disparification and lineage diversification lend more support for one of the models described above?

As a case study, we use the Australian plant genus *Hakea* Schrad. & J.C.Wendl. (Proteaceae), a genus of 152 species of woody shrubs and small trees, with a center of diversity in the Mediterranean‐climate region of Southwest Australia, one of the world's most diverse temperate ecoregions. Here, closely related species co‐occur in high numbers at local spatial scales (Thiele and Prober 2014) across predominantly oligotrophic sandy soils, providing a good opportunity to look at how biotic interactions shape biodiversity patterns. *Hakea* has a well‐sampled species‐level phylogeny (Cardillo et al. 2017) and phenotypic database (Skeels and Cardillo 2019a). Floral trait diversity in this genus suggests a potentially important role of floral evolution in the clade's history as *Hakea* exhibits a diverse range of pollination strategies and floral characters (Hanley et al. 2009), with repeated shifts between major animal pollination syndromes (Mast et al. 2012). A predominantly sympatric mode of speciation has been inferred in the group based on phylogenetic and geographic patterns (Skeels and Cardillo 2019b).

Investigating patterns within a monophyletic clade has several advantages over an analysis of geographically defined assemblages. First, evolutionary dynamics can be investigated in the context of well‐established phylogenetic diversification models, providing a robust framework for hypothesis testing. Second, ecological similarity is often predicted by phylogeny and therefore closely related species are strong candidates for a role of interspecific interactions on evolutionary dynamics. Finally, ecological and morphological trait data are more accessible for some well‐studied taxa, such as *Hakea*.

## Materials and Methods

All analyses were done using the R software version 3.5.3 (R Core Team 2013).

### DATA COLLECTION

The phylogeny we use in this study is a dated species tree constructed from phylogenomic data (Cardillo et al. 2017). We placed five species that were present in the survey data but missing in the phylogeny (*H. lasiocarpha*, *H. circumulata*, *H. psilorryncha*, *H. gilbertii*, and *H. subsulcata*) as polytomies at the node of the most recent common ancestor of all species in the same intrageneric species groupings (Barker et al. 1999). For certain analyses, we pruned the phylogeny to contain only species in the survey data (hereafter sample‐based phylogeny).

**Figure 2:**
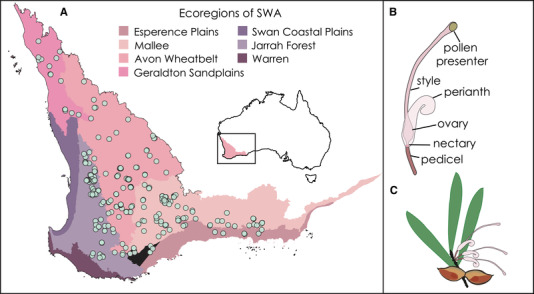
(A) Southwestern Australian floristic survey data from Gibson et al. (2004). (B) Simplified illustration of the floral morphology of a *Hakea* flower, and (C) inflorescence on stem.

Community survey data include all plant species in 400‐m^2^ plots across a large area of Southwestern Australia (Gibson et al. 2004). This scale is below the dispersal distances of plants and pollinators so is appropriate for analyzing pollinator‐mediated species interactions. This dataset includes 52 *Hakea* species from 275 sites (Fig. 2A); 101 sites contained a single Hakea species, whereas diversity in the remaining 174 sites ranged from two to five species, with a mean of 2.5. We obtained morphological and phenological data (start, finish, and duration of the flowering period in months) from Skeels and Cardillo (2019) and Flora of Australia vol. 17B, for these 52 species. We selected the maximum length of the pistil (including the style, stigma, and pollen presenter; Fig. 2B) as a single, widely available morphological trait that best reflects plant‐pollinator interactions, as this organ determines where pollen is collected and deposited during pollination, thereby controlling the mechanics of pollination (Hanley et al. 2009; Lamont et al. 2016). We selected flower color as the trait that reflects pollinator signalling in *Hakea*, as it is readily available from digital photographs, and is correlated with major pollination syndromes (avian vs. insect; Hanley et al. 2009).

### PHOTOGRAPHIC LIBRARY

A rich source of information on phenotypes is digital photographs, and digital photographic libraries are increasingly used in ecological and evolutionary studies (Drury et al. 2019; van der Bijl et al. 2020). For each of the 52 species in the community survey data, we collated a digital photographic library of in bloom inflorescences with images primarily from the Australian Plant Image Index (APII)—a resource managed by the Australian National Botanical Gardens for the identification of the Australian flora (APII 2019), as well as iNaturalist (inaturalist.org) and flickr (flickr.com) when the APII did not have suitable photographs. From this photographic library, we selected between one and three photographs (depending on availability) to extract red, green, and blue (RGB) color data from different organs of the inflorescence. Because different floral organs vary in color and size, the color signal of an inflorescence cannot be attributed to a single value. To account for this, we estimated RGB values of different floral organs (pistil, pollen presenter, nectary, pedicel, and upper and lower perianth separately when they differed) using the image processing software ImageJ (Abràmoff et al. 2004) and recorded the relative proportion of the total color signal of an inflorescence to which that organ contributes.

Where possible, we repeated this across different photographs taken at different times, or with different cameras. Where species did not have multiple photographs, we recorded different flowers within the same photograph. Some species show intraspecific variation in color morphs. We aimed to capture the most representative color morph, rather than capture this variation, and so took RGB measurements that represented the most common morph. This decision was guided by matching colors to those shown in floraBase (Western Australian Herbarium, 2019). In total, we recorded 444 color measurements across the 52 species. We found the color centroids for each organ across different photos for each species to give us a total of 157 measurements. To quantify differences in flower color between species, we obtained pairwise chromatic distances using earth‐mover's distances of color histograms for each species using the colordistance package in R (version 1.1.1; Weller and Westneat 2019). This method considers not only color differences in a color space, but also the proportion of the signal occupied by each color (Weller and Westneat 2019).

Digital images, while being an abundant and valuable resource, are taken under unstandardized light conditions that may affect the RGB values extracted from each photo. To examine the effect of using digital images from online resources, we photographed 11 species under standardized lighting conditions and compared these to online digital photographs from our collated dataset. We also measured UV reflectance for these 11 species to see whether UV plays a key signalling role in *Hakea* (see Appendix S1 for more information).

### FLOWERING PHENOLOGY PATTERNS

To investigate temporal patterns of flowering across Southwestern Australia, we used circular statistics to ask whether the flowering period (months of the year) in Southwestern Australian *Hakea* is distributed unimodally or evenly throughout the year. Our data resolution does not allow us to look at variation in flowering time across the range of species; however, most *Hakea* species in Southwest Australia have small geographic ranges and narrow niche breadths (Skeels and Cardillo 2017) and so are unlikely to show much flowering time variation across their ranges. We transformed months in flower for 52 species into radians and performed a Rayleigh test, which tests for uniformity and periodicity in circular data. We also tested whether species whose geographic ranges overlap also overlap in flowering phenology. Geographic and phenological overlaps were calculated as the proportion of the smaller ranged species that overlaps the larger ranged species and the proportion of the months of the shorter flowering period species that overlaps the longer flowering period species, respectively. We also repeated analyses of community assembly patterns (below) on all communities (*n* = 275) as well as the subset of communities, which contained at least one phenologically overlapping species pair (*n* = 266). We found results to be qualitatively the same so present the results from all communities.

### COMMUNITY ASSEMBLY PATTERNS

We asked whether pairwise morphological differences (pistil length) and earthmover's color distances are more or less similar within communities than expected by chance. To do this, we estimated functional richness (FRic; Villeger et al. 2008), which measures functional diversity as the ratio of the trait‐space volume of each community to the total trait‐space volume of all *Hakea* species across communities. FRic has been shown to be among the best functional diversity metrics to distinguish between community assembly processes when communities contain <10 species (Mouchet et al. 2010) and was calculated on sites with more than two species (39 sites in our dataset). We then asked whether phylogenetic relatedness is more or less similar within communities than expected by chance. We calculated mean pairwise phylogenetic distances (MPD; Webb 2000) as a measure of phylogenetic clustering using the sample‐based phylogeny. To test whether our empirical estimates of FRic and MPD differ from the range of values expected by chance, we simulated community data under two null models of community assembly that shuffle observed species occurrences among sites while approximately maintaining species frequencies and site richness. We used both the independent swap algorithm (Gotelli 2000), which shuffles species among all communities with equal probability, and a dispersal null model (Miller et al. 2017a) in which the probability of a species being assigned to a community is inversely weighted by an exponential function of their geographic distance from the community, thereby accounting for dispersal limitation across broad geographic distances. From 1000 simulated assemblages under each null model, we estimated FRic based on color and morphological distances using the FD package in R (version 1.0; Laliberte et al. 2014), as well as MPD with the picante package (version 1.8.2; Kembel et al. 2010). We then tested whether the mean values of FRic and MPD across communities fall in the upper or lower tail of the simulated distributions.

### MACROEVOLUTIONARY PATTERNS

To look at how floral traits are distributed across lineages, we calculated phylogenetic signal for flower color and pistil length using Blomberg's *K* (Blomberg et al. 2003) and Pagel's *λ* (Pagel 1999) with the phylosig function in the phytools package (version 0.7; Revell 2012). To do this, we first performed principal component analysis on the RGB values of the perianth and pistil colors separately, as well as the dominant color in each species’ inflorescence as taken from the organ that makes up the greatest proportion of the color signal. We then measured *K* and *λ* on the first principal component that accounted for more than 90% of the variation in color for all three measurements. We also calculated *K* and *λ* on the raw values of pistil length. We applied a model selection approach to ask whether the mode of trait evolution was best supported by BM (in which trait evolution follows a pattern of stochastic drift), Ornstein‐Uhlenbeck (OU; in which trait evolution is constrained toward an optimal value), or White Noise (WN; in which the trait evolution is independent of the phylogeny) models of trait evolution in the R package Geiger (version 2.0.7; Harmon et al 2008). We compared the goodness of fit of these models using Akaike information criterion corrected for small sample size (AICc).

To determine the relative timing of diversification of floral traits in *Hakea*, we reconstructed disparity through time (DTT) of flower color and pistil length separately. We calculate DTT following Harmon et al. (2003), using the R package dispRity (version 1.5.0; Guillerme 2018), which measures the mean disparity (mean squared pairwise distances between species) of each subclade measured at each node in the phylogeny compared to the total disparity of the whole clade. We decomposed the color distance matrix into a lower number of dimensions using principal coordinate analysis (*k* = 5) to use as input for DTT, whereas we used the raw measurements of pistil length. We compared the DTT of each floral trait to the DTT distribution from 1000 BM simulations of traits along the sample‐based phylogeny. To complement the DTT analysis, we also constructed lineages through time (LTT) plots (Nee et al. 1992) for both the sample‐based and full phylogeny of *Hakea*. LTTs show the number of reconstructed lineages present at different time points throughout the clade's history.

To gain a better understanding of evolutionary rate heterogeneity and the dynamics between the rate of floral trait evolution and the rate of lineage diversification through time, we estimated branch‐specific values of these rates along the sample‐based phylogeny. We estimated the rate of floral color and pistil length evolution using phylogenetic ridge regression, as implemented in the R package RRPhylo (version 2.5.0; Castiglione et al. 2018). For simplicity, we ran the model on a single RGB color value for each species, chosen as the color making up the largest proportion of the flower. To estimate branch‐specific rates of lineage diversification, we used the ClaDS2 model, which estimates speciation rates across the phylogeny, assuming a constant turnover rate through time, using a Bayesian approach (Maliet et al. 2019) in the R package RPANDA (version 1.9; Morlon et al. 2016). Recent work has raised concerns about nonidentifiability of estimates of speciation and extinction rates from phylogenetic data (Louca and Pennel 2020). The hypothesis‐testing framework of ClaDS2 in some ways circumvents this problem, because it constrains the realm of parameter estimates based on well‐founded prior assumptions about the diversification process; for example, rate shifts are unlikely in very short time periods; rates are correlated across the phylogeny; and extinction rates are variable across the tree but species turnover is constrained (Morlon et al. 2020). We ran the model for 5000 MCMC iterations, with a thinning rate of 10, using the proportion of species in the sample relative to all *Hakea* species to adjust the model for taxon sampling. For further analysis, we extracted the maximum a posteriori estimates for each branch of the phylogeny. We excluded species that were not in the original molecular phylogeny, to avoid any impact their uncertain placement might have on estimating diversification rates.

We then fit a model of how floral trait evolutionary rate is associated with diversification rate across branches of the phylogeny or branch age. We fit a model with gaussian error structure (for flower color and pistil length separately), using log‐transformed trait evolution rate (+0.1 to deal with some near zero estimated rates) as the response and log‐transformed and standardized diversification rate (+0.1) and branch age (distance from the root) as predictors in the model. To account for phylogenetic nonindependence of rates along branches, where rates on phylogenetically nearby branches may be correlated due to shared ancestry, we used a phylogenetic linear mixed model (PGLMM) that allows for data at internal nodes in the phylogeny (an ancestral PGLMM, or APGLMM). We used the implementation of APGLMM in the R package phyr (version 1.1.0; Li et al. 2020) to fit the model (using function pglmm). This implementation uses a Bayesian approach, fit using integrated nested Laplace approximation, and so we examine the 95% credible intervals for inference. Rates along branches were associated with their terminal nodes for the purposes of accounting for phylogenetic structure. Following general recommendations for mixed and other Bayesian models (Lemoine 2019), we chose a weakly informative prior for the strength of the phylogenetic random effect. We based our prior on an estimate of the standard deviation in a BM model applied to the model residuals when the model was fit with a standard linear model regression. For an unbiased estimate of this value, we used the mean of the squared phylogenetic independent contrasts of the residuals. Then, for the prior on the phylogenetic random effect standard deviation, we used an exponential distribution, where 1% of the probability density fell on values greater than three times the unbiased BM estimate. This gives a relatively flat prior, with more probability density at low values, but very little probability density at unrealistically high evolutionary rates, and is the same kind of general weakly informative prior recommended by Simson et al. 2017.

We compared the fit of diversification rate and branch age as predictors using Bayes factors (BF). We used a standard scale to interpret BF, as described in Kass and Raftery (1995), where absolute BF values less than 3.2 indicate more or less equivalent models, 3.2–10 are small to substantial improvements in model fit, and greater than 10 are very strong improvements in fit.

## Results

### FLOWERING SYNCHRONY

**Figure 3:**
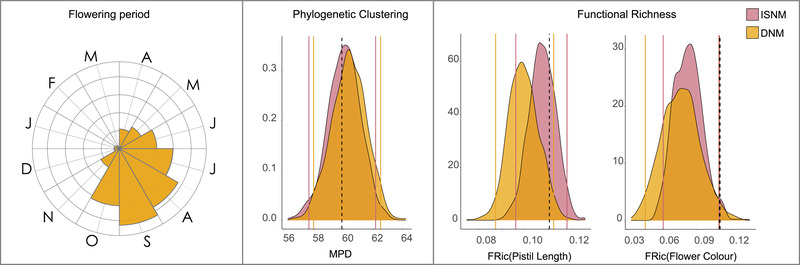
Distribution of flowering times in months for 52 species of *Hakea*, and the observed and null distributions from two models (independent swap [ISNM] and dispersal null model [DNM]) of mean phylogenetic distance (MPD) and functional richness (FRic) measured for two floral traits, maximum pistil length and flower color, averaged across communities in Southwest Australia. Upper and lower 2.5% quantiles indicated with vertical lines of matching color, and the observed metric is given by the vertical dashed black lines.

The 52 Southwest Australian Hakea species in our dataset flower from April to November, but show a unimodal distribution of flowering times, with the greatest number of species co‐flowering in September (Rayleigh test, *z* = 0.555, *P* < 0.001; Fig. 3). We also found that more than 90% of species pairs that co‐occur in ecological communities also overlap in flowering times (172/187 pairwise combinations; Fig. S1) and of 101 sites containing more than a single *Hakea* species, 92 contained species that overlap in their flowering times (91%).

### COMMUNITY ASSEMBLY

The mean FRic of flower color for assemblages across Southwest Australia was greater than the null expectation based on both the independent swap and the dispersal null models (Fig. 3). Mean FRic of pistil length and MPD falls within the 95% confidence intervals of both null distributions and can be considered not significantly different than expected according to either null model. This suggests that across Southwest Australia, communities tend to occupy a greater volume of trait space for flower color than expected if communities across the region were assembled at random.

### EVOLUTION OF FLORAL TRAITS

Pistil color shows phylogenetic signal significantly different from zero (*K* = 0.69, *P* = 0.01; *λ* = 0.54, *P* < 0.001) and both BM and OU models were a better fit than WN (Fig. S2). Significant phylogenetic signal was not detected in the dominant color (*K* = 0.45, *P* = 0.59; *λ* < 0.001, *P* = 1), perianth color (*K* = 0.54, *P* = 0.15; *λ* = 0.41, *P* = 0.24), or pistil length (*K* = 0.60, *P* = 0.053; *λ* = 0.19, *P* = 0.24). All three of these traits showed strongest support for a WN model of trait evolution; however, an OU model could not be rejected (delta AIC < 2). PC1 of the dominant color was correlated with PC1 of perianth color (Pearson's *r* = −0.8) but not with pistil color (*r* = 0.4) and the perianth typically contributed the most to the dominant color of the inflorescence.

**Figure 4:**
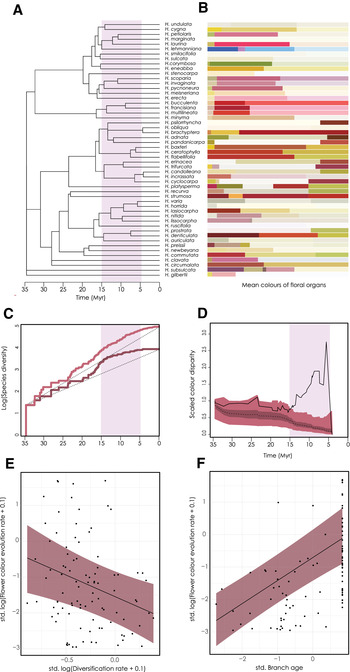
Evolution of disparity in flower color. (A) Phylogeny of 52 species from across Southwest Australia, sampled in the community survey data. (B) Average colors sampled from each species for five different floral organs, with the size relative to the contributed proportion of the inflorescence color signal. (C) Lineages through time plots of log‐transformed species diversity. The light pink is the LTT of the full phylogeny (135 species), and in dark purple is the sample‐based phylogeny (52 species). Dotted lines show the null expectation under a constant rates model of lineage diversification. (D) Disparity through time plot of flower color with disparity measured as the mean squared pairwise distances. Red polygons show the expectation under a Brownian motion model from 1000 simulations, with the dotted line showing the mean disparity from these simulations. The black line shows the estimated disparity for flower color. The relationship between the estimated per branch rates of diversification and flower color evolution (E) and branch age (F) from ancestral phylogenetic generalized linear mixed model (APGLMM) with rates (+0.1) plotted on a log scale.

DTT analysis showed that subclade disparity was mostly consistent with that expected under BM for pistil length (Fig. S3). Flower color, on the other hand, shows subclade disparity consistent with that expected under BM for much of this early history (Fig. 4D), followed by an extended period, from roughly 15 to 5 million years ago, characterized by significant deviation away from this null expectation (Fig. 4D). Large values show that within‐clade variation is high relative to between‐clade variation, suggesting that lineages that originated during this later period have highly divergent color morphs and can explain a significant proportion of the total color variation across the genus. We also see relatively high pairwise color distances at low phylogenetic distances (Fig. S4), which is inconsistent with BM (Cadotte et al. 2017). This relatively late period of radiation is also characterized by a slowdown in lineage diversification. These diversification dynamics are seen in both the full and sample‐based phylogenies (Fig. 4C).

The regression models of evolutionary rates revealed that there was generally a negative relationship between diversification rate and flower color evolution rate (slope = −1.21, CI = [−2.03, −0.37]; Fig. 4E), such that the slower estimated diversification is, the faster estimated color change tends to be along phylogenetic branches. Flower color evolution rate was also credibly positively related to branch age (slope = 0.71, CI = [0.50, 0.92]; Fig. 4F), such that older branches tend to have slower rates of flower color evolution. A model of flower color evolution rate including branch age as a covariate with diversification rate was a substantially better fit than a model including just diversification rate (BF = 9.7), but a model with branch age as a single response was slightly better than model with branch age and diversification rate (BF = 3.6). Additionally, the model with branch age only was considerably better than an intercept only model (BF = 13.1), but the diversification only model was no better (BF = 0.20). In contrast, rates of pistil length evolution did not show any credible relationship with diversification rate (slope = −0.16, CI = [−0.70, 0.40]; Fig S5A) and only very slightly with branch age (slope = 0.18, CI = [0.028, 0.33]; Fig. S5C). Pistil length models all performed worse than an intercept‐only model (age‐only model: BF = −3.1; diversification‐only model: BF = −4.6; both: BF = −6.9).

## Discussion

Mediterranean‐climate shrubland ecosystems are a challenge for ecologists because it is not clear how multiple closely related plant species can coexist in such low‐productivity environments, often with very little habitat or topographic heterogeneity, without negative effects of reproductive interference and pollinator competition. *Hakea* is one of the most diverse plant genera in the Southwest Australian biodiversity hotspot, and using readily available trait data and digital photographs, our results demonstrate that species in the genus have not shifted their reproductive phenology to reduce local flowering synchrony. Instead, the assembly of these shrubland plant communities can be explained by local divergence in floral phenotypes, and in particular, flower color. Together, macroevolutionary patterns of diversification and patterns of ecological sorting across present‐day communities suggest that negative biotic interactions, such as pollinator competition, may play a key role in both driving the evolution of flower color and facilitating the coexistence of species.

### FLORAL TRAITS IN SPECIES‐RICH ECOLOGICAL COMMUNITIES

Floral traits have been shown to be strongly associated with pollinator preferences, and differences in size, color, pattern, or scent of a floral display act as signals to the behavioral and sensory ecology of animal pollinators (Schiestl and Johnson 2013). Different colors of flowers have repeatedly evolved as a signal to the different optical systems of animal pollinators (Rodríguez‐Gironés and Santamaría 2004). In some cases, whole communities converge on similar flower color signals to facilitate the attraction of mutual pollinators (Kantsa et al. 2017). In other cases, species in communities show divergent flower color signals to reduce competition or reproductive interference with closely related species (McEwen and Vamosi 2010). The average volume of trait space for flower color across ecological communities in Southwestern Australian *Hakea* supports this latter result; that nonrandom structure of floral traits in ecological communities is due to diversity, not uniformity, in floral displays.

High FRic of floral phenotypes in ecological communities of Southwest Australia may promote pollinator diversity while ensuring greater fidelity of pollen flow between individuals of the same species. This is because many pollinator species that rely on floral resources are tuned to pay attention to specific cues from flowers such that they are more likely to visit certain species (Pauw 2013, 2019). In *Hakea*, one of the most striking distinctions between floral phenotypes is that of avian compared to insect‐pollinated species (Hanley et al. 2009). Typical of many plant species, red flowers are commonly associated with an avian pollination mode, whereas insect‐pollinated flowers are more typically yellow, white, or cream in color (Grant 1966; Ford et al. 1979; Rodríguez‐Gironés and Santamaría 2004). *Hakea* species are known to be pollinated by different orders of insects including Diptera, Lepidoptera, and Hymenoptera (Barker et al. 1999), which each have been recorded to show a degree of preference for particular color morphs in Southwest Australia (Groom and Lamont 2015). Although the actual specificity of *Hakea*’s pollination vectors is largely unknown, the observation of greater than expected FRic of flower color suggests that alternate pollination syndromes may facilitate co‐occurrence by reducing niche overlap—a prediction that can be tested empirically in future studies.

### FLORAL MACROEVOLUTION

The tempo and mode of flower color evolution, but not morphological evolution, is more consistent with a negative interactions model than with a speciational or allopatric divergence model (Fig. 1), for several reasons. First, low phylogenetic signal in dominant flower color and a lack of significant phylogenetic structure of Hakea communities are consistent with a key role for flower color diversity in structuring communities, or at least with the lack of any important role for other, phylogenetically conserved traits. The negative interactions model of trait evolution is diversity dependent; as species diversity increases in a region, the number of potential negative interactions increases, which in turn is expected to increase the rate of disparification of ecological traits that mediate negative interactions (Skeels and Cardillo 2019a). A positive relationship between rates of flower color evolution and branch age (Fig. S4F) supports this, and shows that rates of flower color evolution have been increasing toward the present‐day in concert with increasing standing diversity of Southwest Australia. Flower color evolution rates are also negatively related with diversification rates (Fig. 4). However, this is likely due to covariation between diversification rates and branch age (Fig. S5), as diversification rates do not provide a credibly better fit than an intercept‐only model, and we observe a strong pattern of lineage diversification slowdown after 15 million years ago (Fig. 4). Together these results suggest negative diversity‐dependent processes and increased competition resulting from a higher density of interacting species (Phillimore and Price 2008; Rabosky 2013).

Previous studies have found a positive relationship between species diversity and floral divergence in sympatric lineages, suggesting diversity dependence is important, but so far have been unable to distinguish between the speciational and negative interactions models of floral divergence (Armbruster and Muchhala 2009; Weber et al. 2016). Our result goes some way to clarifying this in *Hakea*; if floral divergence was involved in facilitating speciation, then we would expect a positive, rather than negative, relationship between rates of lineage diversification and floral trait disparification. The negative interactions model predicts a negative relationship between lineage diversification and trait disparification rates, because both are driven by a third covariate—competition. Because floral trait disparification is negatively related to lineage diversification, and because branch age (and therefore species diversity, a proxy for interspecific competition) is a better predictor of trait disparification rates, we propose that high species diversity in *Hakea* has driven floral disparification.

The evidence in support of competition‐driven floral evolution goes against the long‐standing idea that shifts in floral traits are associated with rapid diversification dynamics in angiosperms. Although some studies have found positive associations between the presence of floral traits like zygomorphic flowers or nectar spurs and rates of diversification across clades (e.g., O'Meara et al. 2016), far fewer studies have investigated the link between rates of floral trait divergence and lineage diversification within clades that share a common flower type. Our study is one of the first to highlight how the diversity in key floral traits has evolved in the later stages of a radiation when species diversity is already high. This supports a “beta‐first” model of diversification (Diamond and Case 1986; Skeels and Cardillo 2019a): early diversification is coupled with trait divergence along environmental and habitat niche‐axes (β‐traits), whereas later stages of diversification are associated with the evolution of traits that mediate competition between sympatric species (α‐traits; Silvertown et al. 2006). Competitive pressure for ecological divergence does not seem to be limited to floral traits; however, other key ecological traits, notably seed mass, which determines the timing and success of germination, have also been shown to diversify rapidly late in the radiation of *Hakea* (Skeels and Cardillo 2019a) and there is support for a predominantly sympatric mode of speciation in the genus (Skeels and Cardillo 2019b). Taken together, these studies suggest that a process of competition‐driven ecological differentiation has been key to the development of today's high‐diversity assemblages in *Hakea*.

If elevated rates of *Hakea* flower color evolution in the mid to late Miocene (15–5 million years ago; Fig. 4D) were a response to increasing species density and niche‐space saturation, one of the factors mediating flower color divergence may have been the major group of avian pollinators of *Hakea*, Meliphagid honeyeaters (Hopper 1981; Groom and Lamont 2015). The meliphagids originated in the late Oligocene (Marki et al. 2017) and radiated into a range of ecological niches during the mid‐late Miocene (Marki et al. 2019), a period when aridification and widespread emergence of open habitats spurred the diversification of much of Australia's heavy nectar‐producing flora, including many lineages of Proteaceae (Onstein et al. 2016). Ecologically divergent honeyeaters regularly co‐occur in high numbers (Miller et al. 2017b; Marki et al. 2019), yet although some species may specialize on particular nectar sources (Collins and Rebelo 1987; Pauw 2019), most avian pollinators in Australia are generalist and will take nectar from a number of different species (Ford et al. 1979). It remains unclear whether the radiation of honeyeaters during this period may have provided ecological opportunity for pollination niche divergence in *Hakea*. An alternative is that shifts between insect and avian pollination syndromes, estimated to have occurred many times throughout the radiation of *Hakea* (Mast et al. 2012), provide a clearer pathway to floral niche partitioning than niche partitioning between species within the avian syndrome.

Flower color may also evolve in response to nonpollinator‐mediated selection (Irwin et al. 2003; Strauss and Whittall 2006), for example, anthocyanin pigments are linked with pink‐red flower color but may also confer tolerance to stressful environmental conditions such as drought or heat (Warren and Mackenzie 2001; Strauss and Whittall 2006). Furthermore, anthocyanins may be present in floral tissue as an indirect consequence of selection for anthocyanins in other vegetative tissues (Armbruster 2002). Aridification of Australia during the Neogene could therefore provide an alternative mechanism for increasing rates of flower color evolution during the radiation of *Hakea*: increasing temperatures and seasonality of Mediterranean‐climate ecosystems in Southwest Australia (Lamont and He 2017) and associated drought or other abiotic stresses led to selection for increased anthocyanin production. We cannot rule this out in *Hakea*; however, adaptations to abiotic selection pressures would be expected to produce either random patterns of ecological sorting of floral traits or a pattern of convergence in communities as species are exposed to the same selection pressures. Although ecological sorting of flower color shows the opposite pattern, it would be interesting to look at the distribution of flower color along the steep temperature and precipitation gradients of Southwest Australia.

## Conclusion

Our combined results from community assembly and macroevolutionary analyses support the idea that negative interactions have played a role in shaping the evolution and divergence of floral traits in *Hakea*. As diversity increases within a community, selective pressure increases for species to become phenotypically and ecologically divergent from their sympatric relatives (Armbruster and Muchhala 2009; Weber and Strauss 2016). These interactions also play out over much deeper timescales and we see that as diversity increased within Southwest Australia, so too did the rate of phenotypic divergence for an important floral trait—flower color. By analyzing diversity‐dependent processes at both ecological and macroevolutionary scales in the same framework, our study adds to a growing body of evidence that ecological interactions can shape macroevolutionary patterns, highlighting the importance of considering the ecological context of diversification (Weber et al. 2017; Skeels and Cardillo 2019a).

Our method of using abundant and accessible digital photographic data offers an opportunity to increase the taxonomic and spatial scope of pollination studies in the future, for example, to understand the degree to which species from different taxa interact via pollination vectors in high‐diversity ecosystems. However, many species are still poorly sampled in digital photographic databases, and we often lack information of important floral traits (e.g., nectar production, scent) as well as phenotypic polymorphisms that may mediate competitive interactions and provide further insight into character displacement and the speciation process (Li et al. 2018). Nonetheless, we found that strong ecological and evolutionary signals present in key, readily available traits can be used to tease apart different hypotheses for the evolution of floral disparity.

## AUTHOR CONTRIBUTIONS

AS, RD, and IM designed the study. AS and IM collected the data. AS and RD analyzed the data. AS wrote the first draft of the manuscript and all authors made significant contributions to revisions.

## CONFLICT OF INTEREST

The authors declare no conflict of interest.

Associate Editor: S. Wright

## Supporting information




**Appendix S1**. Color standardization and UV reflectance

**Figure S1**. Heatmap of flowering times overlap between species of *Hakea*.

**Figure S2**. Akaike weights of white noise, Brownian motion, and Ornstein‐Uhlenbeck models of trait evolution for the first principal component of the RGB values of the dominant flower color, the perianth color, and the pistil color, as well as the length of the pistil.

**Figure S3**. Disparity through time plot for maximum pistil length. Disparity measured as the mean squared pairwise distances.

**Figure S4**. The relationship between species pairwise phylogenetic distance and earth mover's flower color distance.

**Figure S5**. The relationship between (a) the estimated per branch rates of pistil length evolution and the estimated per branch rates of diversification, (b) the estimated per branch rates of diversification and the age of each branch, and (c) the estimated per branch rates of pistil length evolution and the age of each branch.

Supporting Information

## Data Availability

All necessary data and R Code to replicate these analyses are made available online (Appendix S2.rar).
