# Supplementary material for: Ecological interactions shape the evolution of flower color in communities across a temperate biodiversity hotspot
Source: Evol Lett. 2021 Apr 28;5(3):277–89. doi: 10.1002/evl3.225 (PMC8190448; doi:10.1002/evl3.225)
Supplement: Supplementary file 1 — Appendix S1. Color standardization and UV reflectance [file EVL3-5-277-s004.docx]

## Supporting Information

## Appendix S1. Colour standardisation and UV reflectance

Digital images, while being an abundant and valuable resource, are taken under unstandardized light conditions which may affect the RGB values extracted from each photo. To see what the effect of using digital images from online resources, rather than taken under standardised conditions, we took photographs of the flowers of eleven *Hakea* species under standardised light conditions. We took raw photographs using a Canon 500D digital SLR with a full spectrum fused-silica conversion (Camera Clinic, Melbourne) and a Canon 100 mm macro lens. The lens was fitted with one of two different filters: UVB (320-400 nm) and visible (400-700 nm). For standardised illumination conditions we adjusted manual parameters to allow only light from a flash unit with UV and visible spectral power distribution. We used a constant camera height and all photographs were taken in the same place and using a spectralon white standard (LabSphere, NH, USA).

We compared our standardised photographs in the visible range (400 – 700nm) with photographs taken from the same online resources as for our photographic library (namely the APII) sampling colours in the same way as described above. To compare images, we performed a principal component analysis on the RGB values of each organ. We compared PC1, which explained more than 91% of the variation in colour data, for the colour of each floral organ from our standardised photographic data to the value of PC1 for the same floral organ of the digital photographs from the APII and found a very strong positive correlation (Pearson’s r = 0.89) suggesting digital images from the APII give very similar colour measurements to those measured under common, standardised conditions.

This study focuses on the visible RGB light spectrum, however many animals including many insect pollinators see ultra-violet light and flowers often attract pollinators using these, rather than visible light wavelengths. To test whether *Hakea* species might be reflecting UV light as a signal to pollinators, we measured UV reflectance measurements of a sample eleven *Hakea* species, including both tentatively insect and bird pollinated species. We adjusted the white balance of standardised images using the spectralon standard, which has high UV reflectance. In the UV photographs there was no reflection of light in any part of the flowers (e.g. flowers were completely black, R=0, G=0, B=0). We found that no *Hakea* flowers reflected UV to a significant degree (i.e., detectable under our light conditions), suggesting that UV signalling is unlikely to play a role in the species sampled.


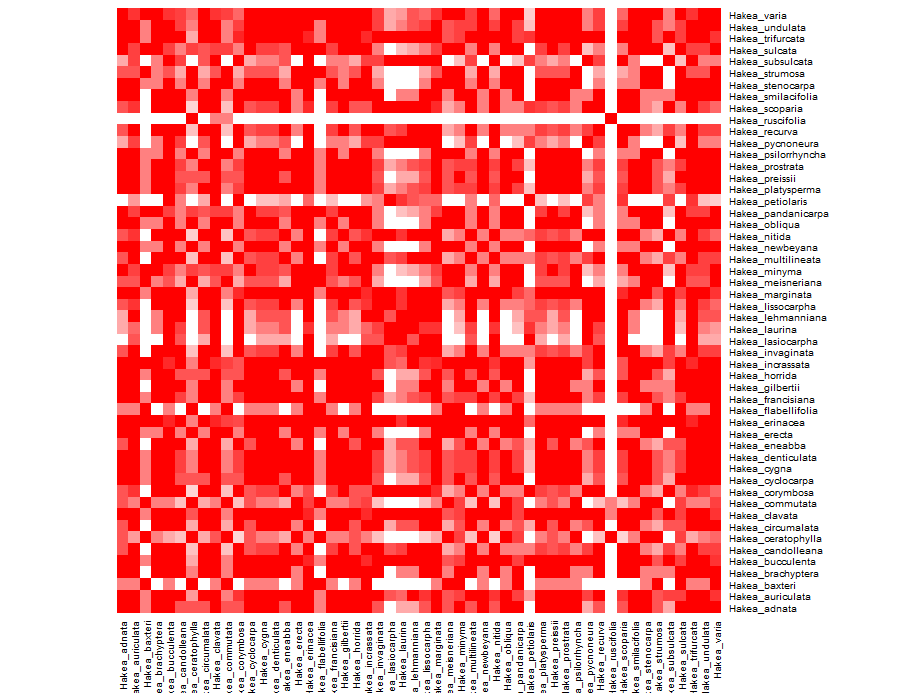


**Figure S1**. Heatmap of flowering times overlap between species of *Hakea*. Gradient of white to red represents no overlap to complete overlap in flowering times of species.


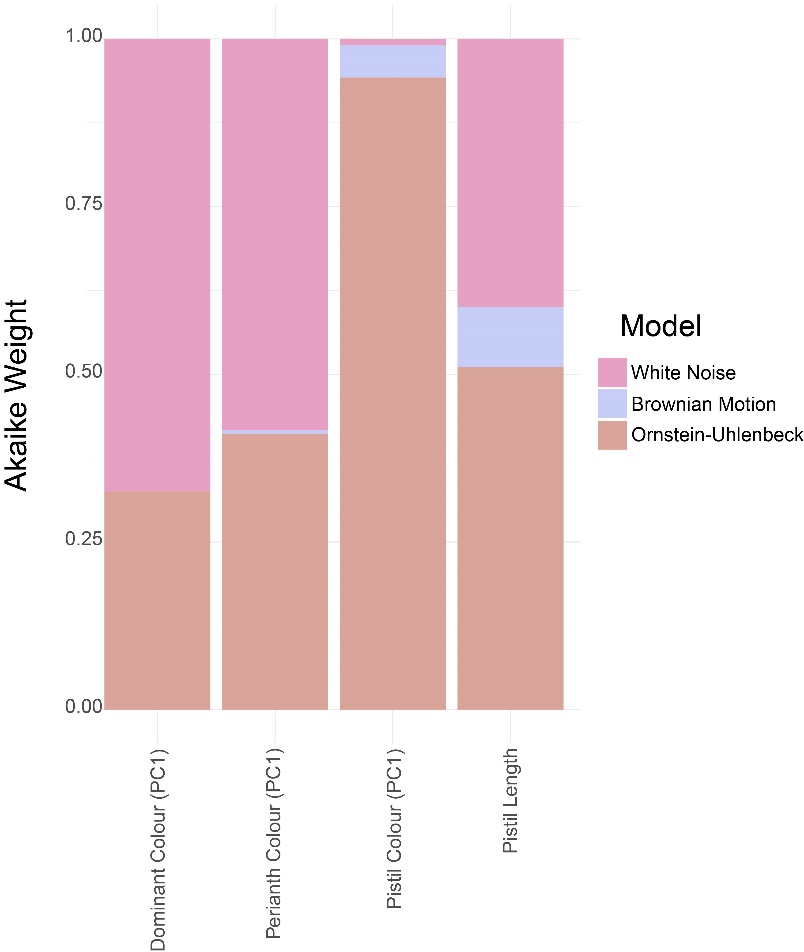


**Figure S2.** Akaike weights of white noise, Brownian motion, and Ornstein-Uhlenck models of trait evolution for the first principal component of the RGB values of the dominant flower colour, the perianth colour, and the pistil colour, as well as the length of the pistil.

**
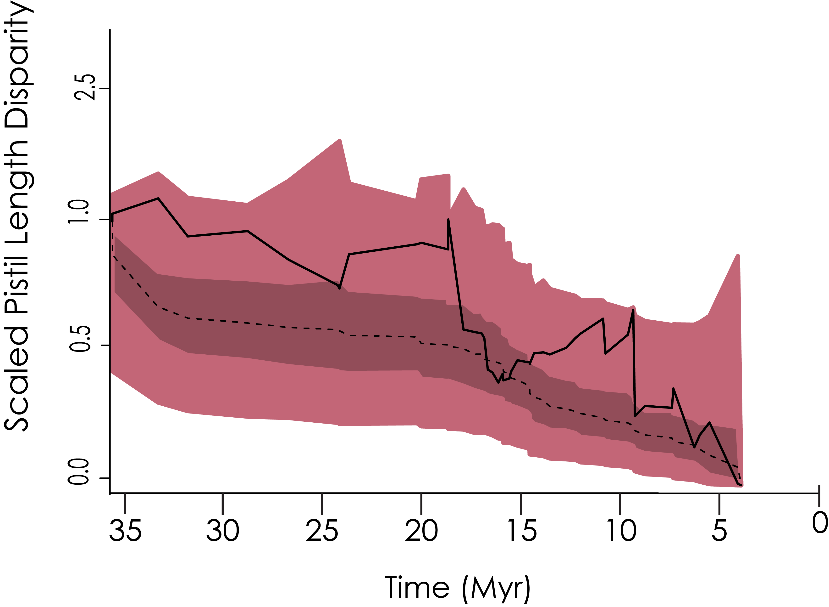
**

**Figure S3** Disparity through time plot for maximum pistil length. Disparity measured as the mean squared pairwise distances. Red polygons show the expectation under Brownian Motion from 1000 simulations, with the dotted line showing the mean disparity from these simulations. The black line shows the estimated disparity for pistil length which falls within the Brownian expectation.


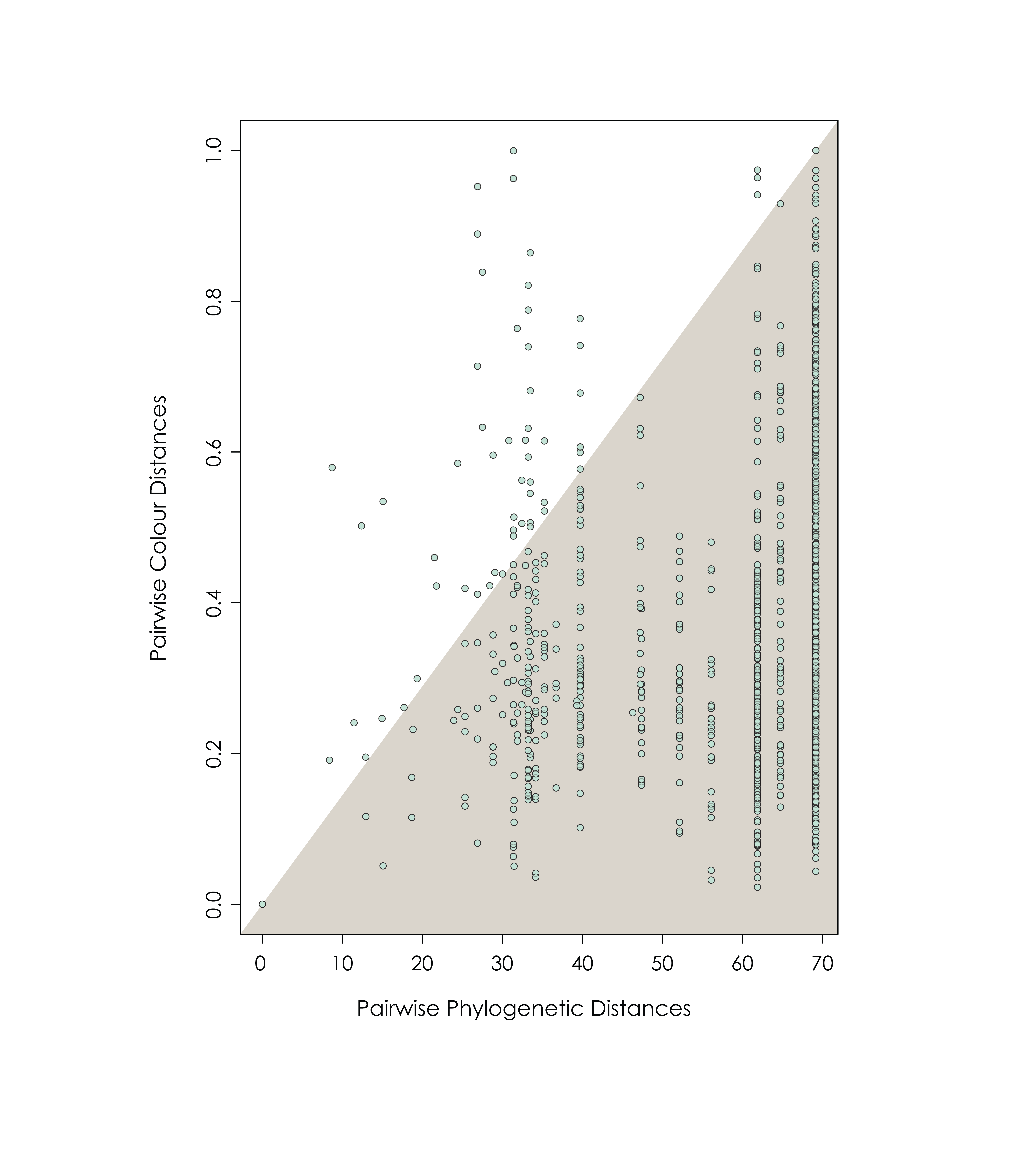


**Figure S4** The relationship between species pairwise phylogenetic distance and earth mover’s flower colour distance. Under Brownian motion we expect a wedge-shaped pattern (brown triangle).

**
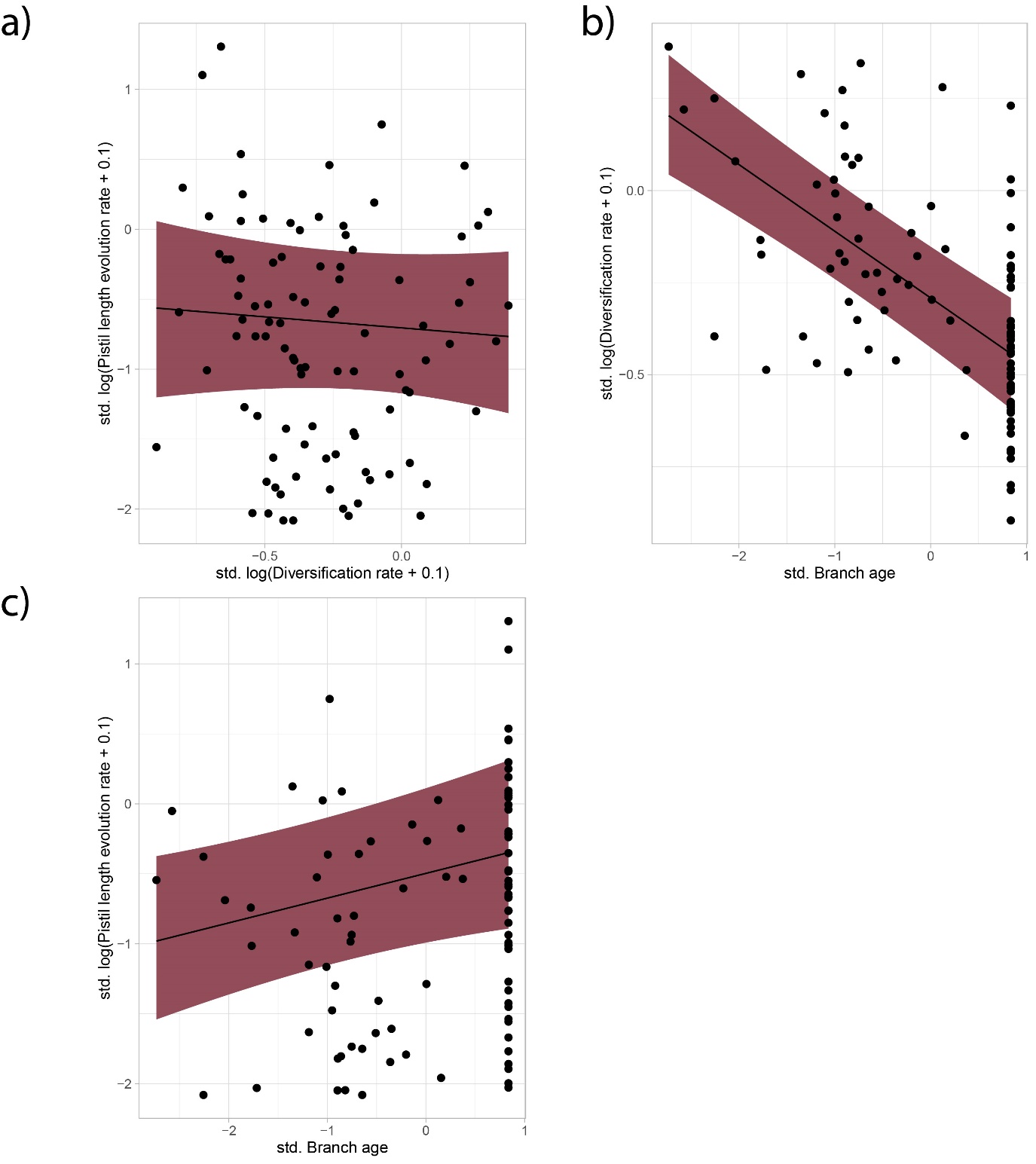
Figure S5** The relationship between a) the estimated per branch rates of pistil length evolution and the estimated per branch rates of diversification, b) the estimated per branch rates of diversification and the age of each branch, c) the estimated per branch rates of pistil length evolution and the age of each branch.
