# Supplementary material for: Ecological interactions shape the evolution of flower color in communities across a temperate biodiversity hotspot
Source: Evol Lett. 2021 Apr 28;5(3):277–89. doi: 10.1002/evl3.225 (PMC8190448; doi:10.1002/evl3.225)
Supplement: Supplementary file 2 — Figure S1. Heatmap of flowering times overlap between species of Hakea. [file EVL3-5-277-s006.pdf]

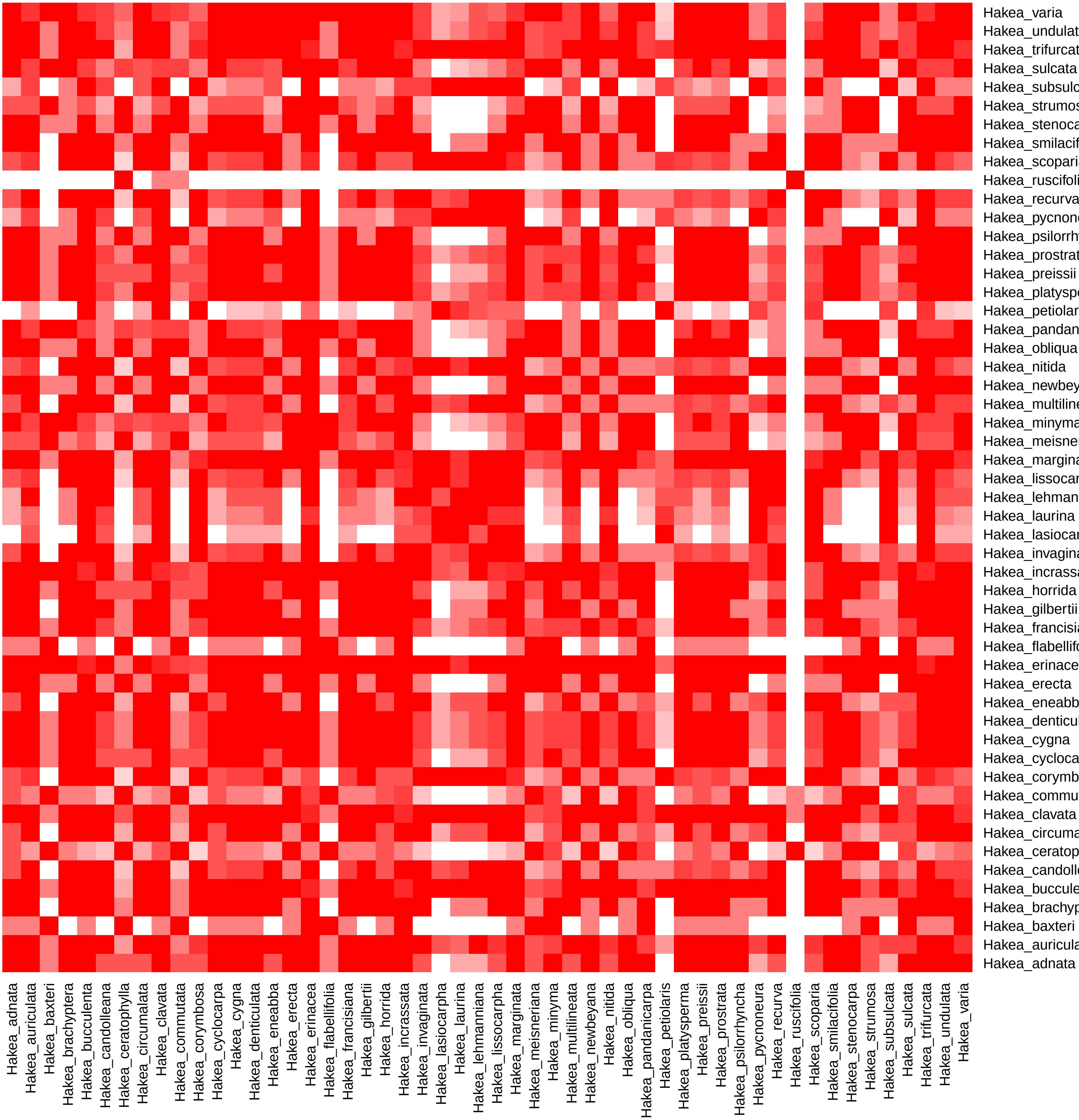
Hakea_varia
Hakea_undulat
Hakea_trifurcat
Hakea_sulcata
Hakea_subsulc
Hakea_strumos
Hakea_stenoca
Hakea_smilacif
Hakea_scopari
Hakea_ruscifoli
Hakea_recurva
Hakea_pycnon
Hakea_psilorrh
Hakea_prostrat
Hakea_preissii
Hakea_platysp
Hakea_petiolar
Hakea_pandan
Hakea_obliqua
Hakea_nitida
Hakea_newbey
Hakea_multiline
Hakea_minyma
Hakea_meisner
Hakea_margina
Hakea_lissocar
Hakea_lehman
Hakea_laurina
Hakea_lasiocar
Hakea_invagina
Hakea_incrass
Hakea_horrida
Hakea_gilbertii
Hakea_francisia
Hakea_flabellif
Hakea_erinace
Hakea_erecta
Hakea_eneabb
Hakea_denticul
Hakea_cygna
Hakea_cycloca
Hakea_corymb
Hakea_commu
Hakea_clavata
Hakea_circuma
Hakea_ceratop
Hakea_candoll
Hakea_buccule
Hakea_brachyp
Hakea_baxteri
Hakea_auricula
Hakea_adnata
Hakea_adnata
Hakea_auriculata
Hakea_baxteri
Hakea_brachyptera
Hakea_bucculenta
Hakea_candolleana
Hakea_ceratophylla
Hakea_circumalata
Hakea_clavata
Hakea_commutata
Hakea_corymbosa
Hakea_cyclocarpa
Hakea_cygna
Hakea_denticulata
Hakea_eneabba
Hakea_erecta
Hakea_erinacea
Hakea_flabellifolia
Hakea_francisiana
Hakea_gilbertii
Hakea_horrida
Hakea_incrassata
Hakea_invaginata
Hakea_lasiocarpha
Hakea_laurina
Hakea_lehmanniana
Hakea_lissocarpha
Hakea_marginata
Hakea_meisneriana
Hakea_minyma
Hakea_multilineata
Hakea_newbeyana
Hakea_nitida
Hakea_obliqua
Hakea_pandanicarpa
Hakea_petiolaris
Hakea_platysperma
Hakea_preissii
Hakea_prostrata
Hakea_psilorrhyncha
Hakea_pycnoneura
Hakea_recurva
Hakea_ruscifolia
Hakea_scoparia
Hakea_smilacifolia
Hakea_stenocarpa
Hakea_strumosa
Hakea_subsulcata
Hakea_sulcata
Hakea_trifurcata
Hakea_undulata
Hakea_varia
