# Supplementary figures and images for: Ecological interactions shape the evolution of flower color in communities across a temperate biodiversity hotspot
Source: Evol Lett. 2021 Apr 28;5(3):277–89. doi: 10.1002/evl3.225 (PMC8190448; doi:10.1002/evl3.225)

# Akaike Weight

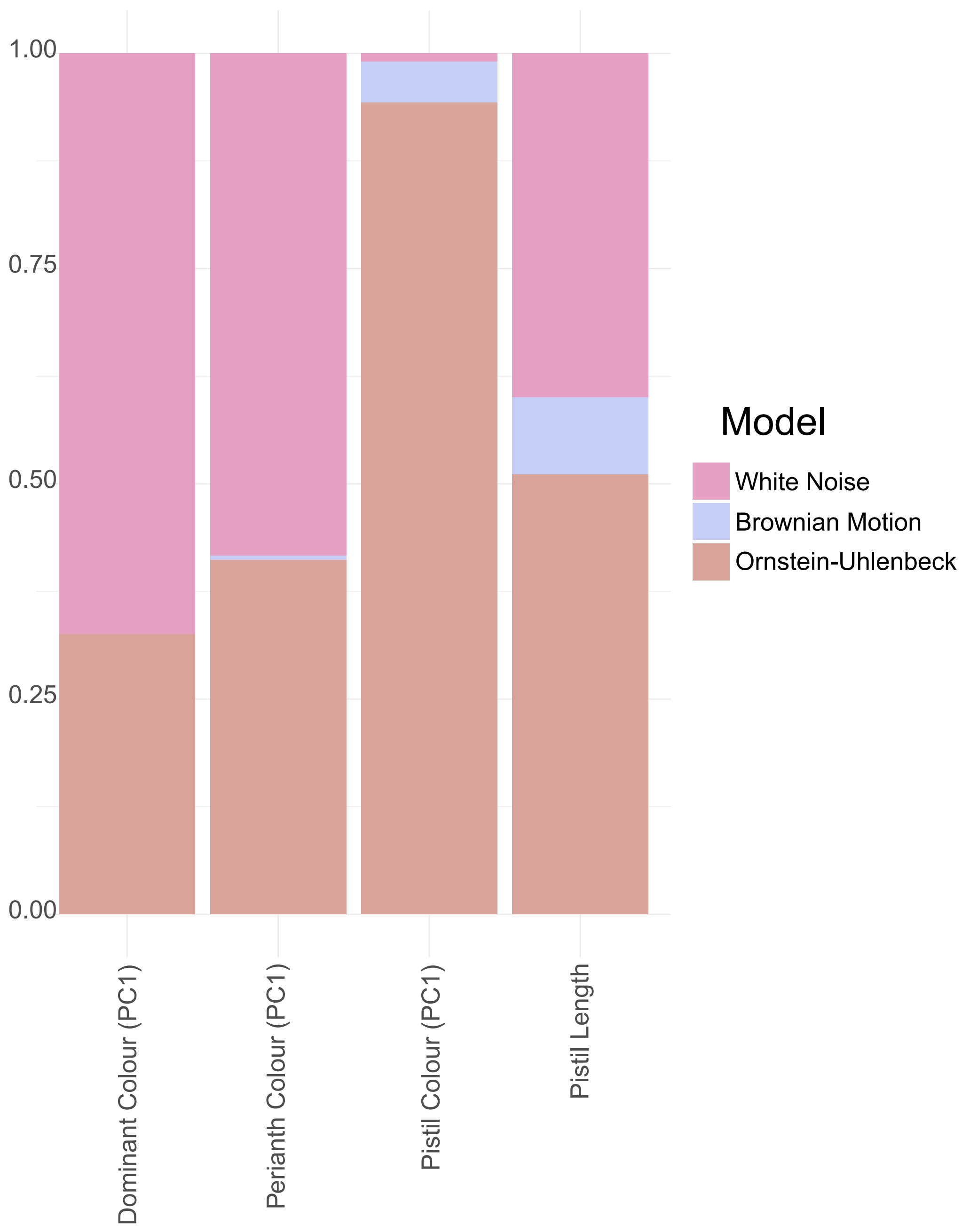

Supplement: Supplementary file 3 — Figure S2. Akaike weights of white noise, Brownian motion, and Ornstein‐Uhlenbeck models of trait evolution for the first principal component of the RGB values of the dominant flower color, the perianth color, and the pistil color, as well as the length of the pistil. [file EVL3-5-277-s003.pdf]

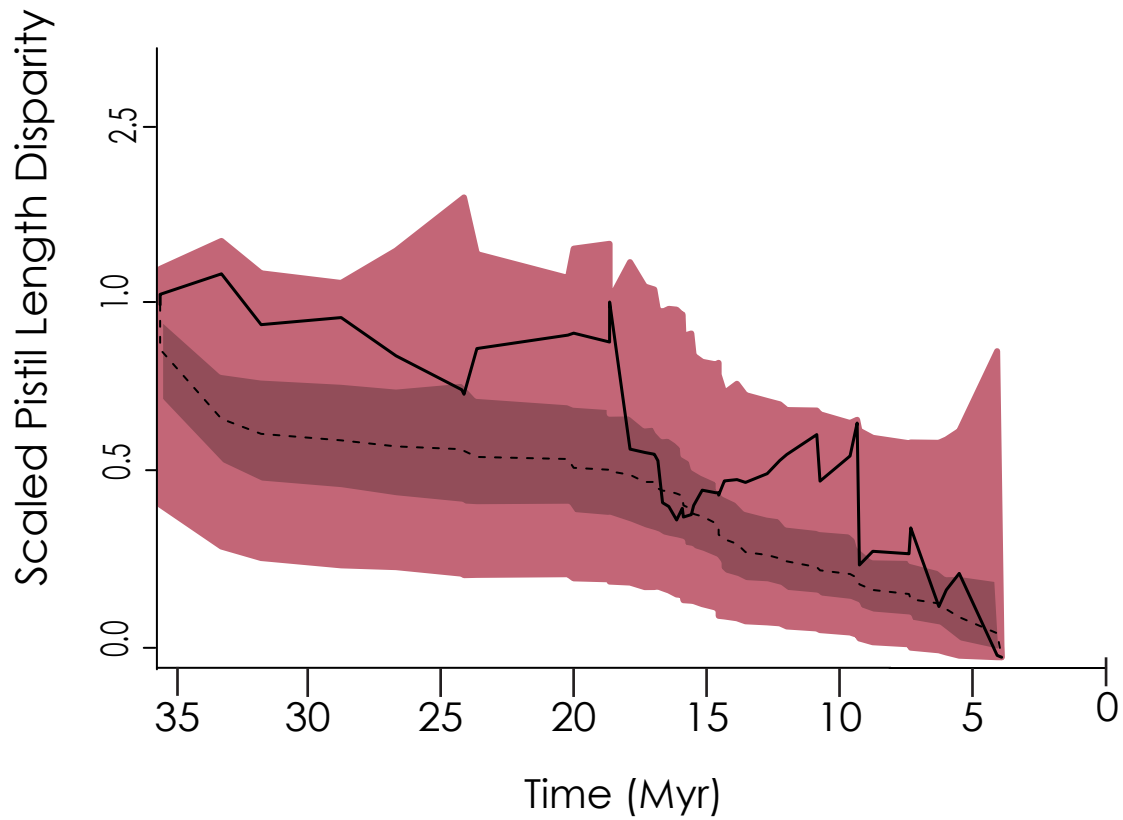

Supplement: Supplementary file 4 — Figure S3. Disparity through time plot for maximum pistil length. Disparity measured as the mean squared pairwise distances. [file EVL3-5-277-s001.pdf]

Pairwise Colour Distances

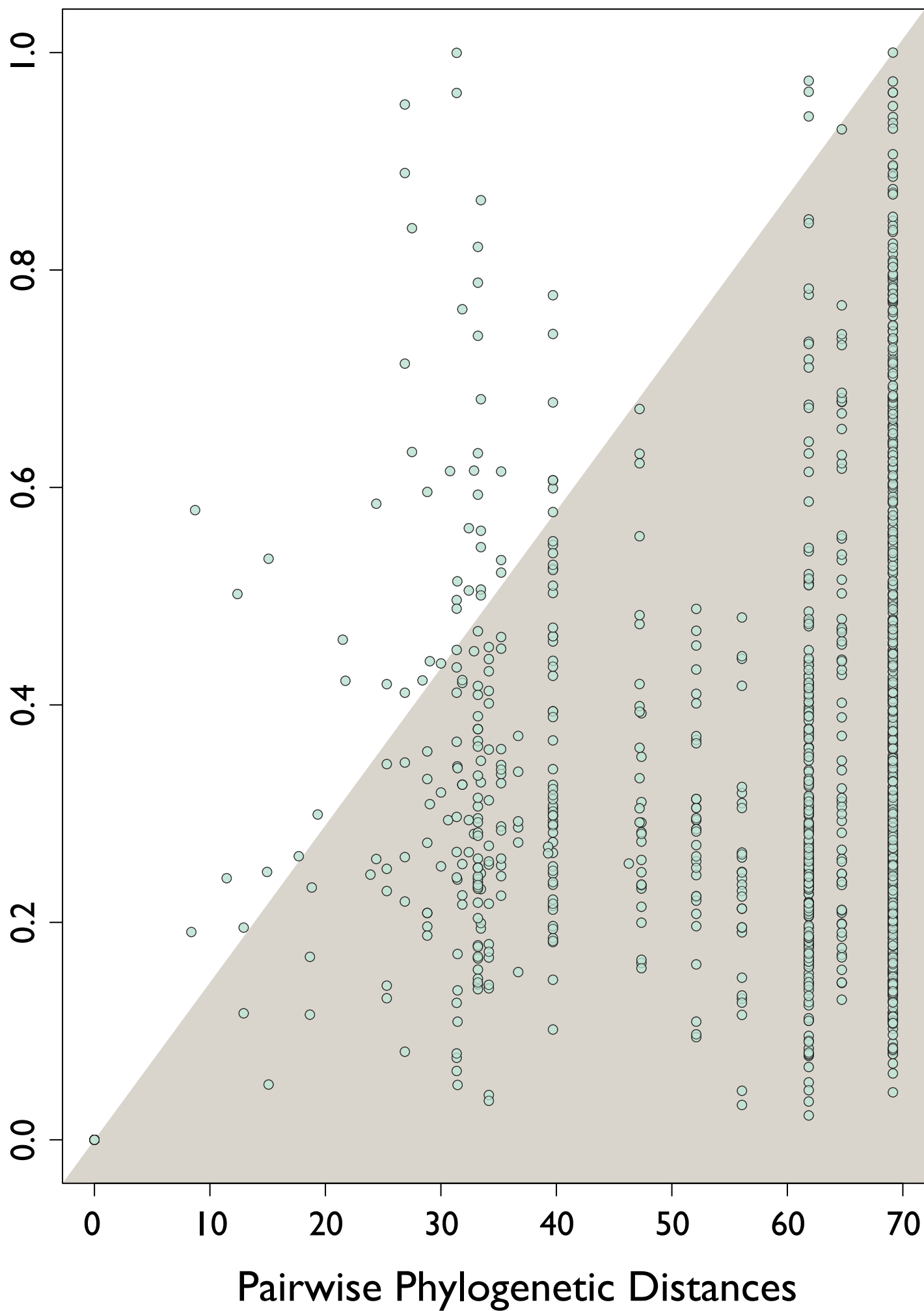

Supplement: Supplementary file 5 — Figure S4. The relationship between species pairwise phylogenetic distance and earth mover's flower color distance. [file EVL3-5-277-s007.pdf]

a)

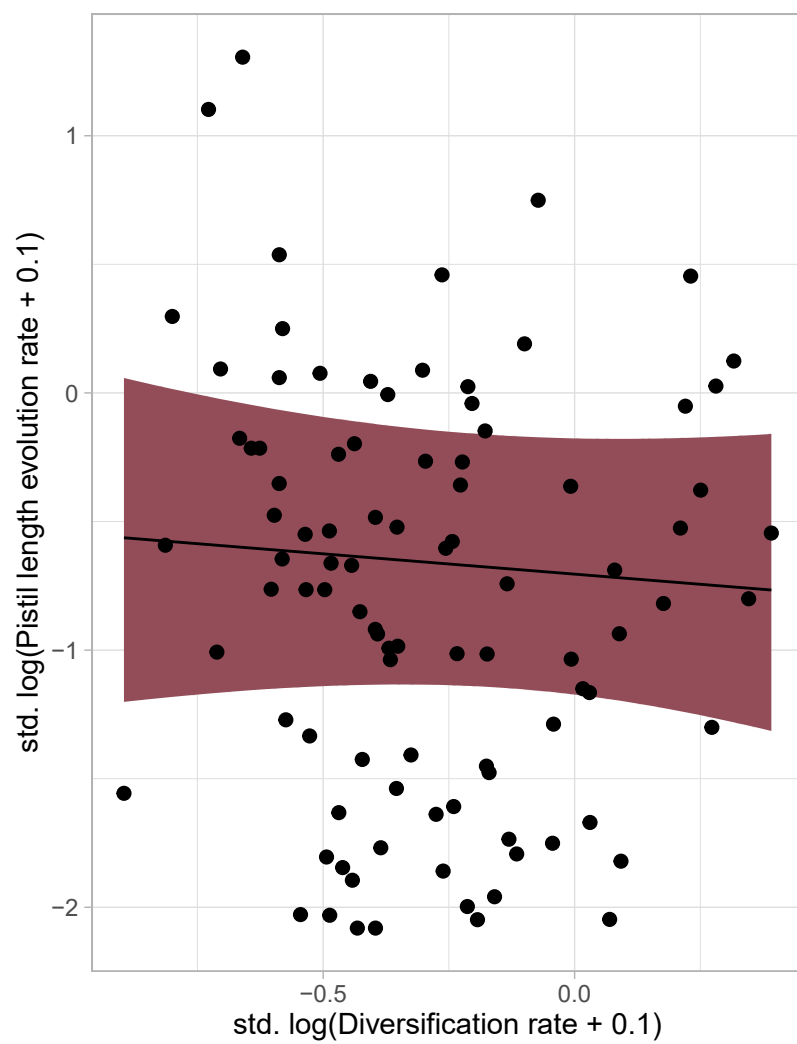

b)

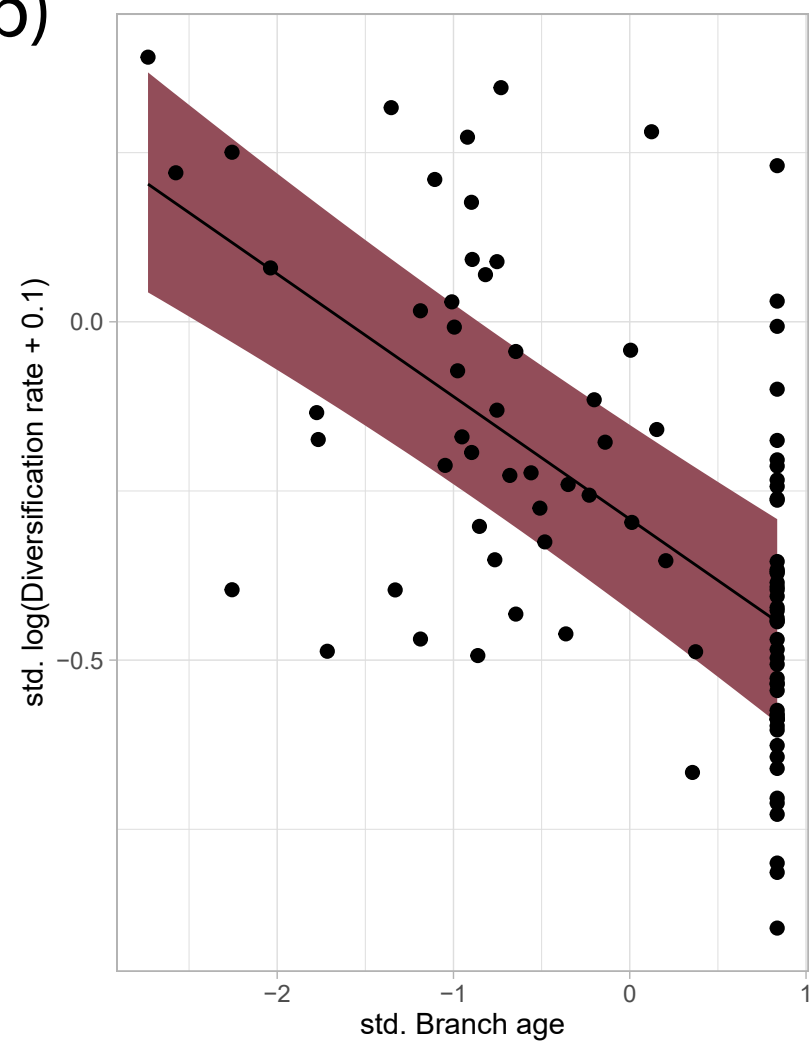

c)

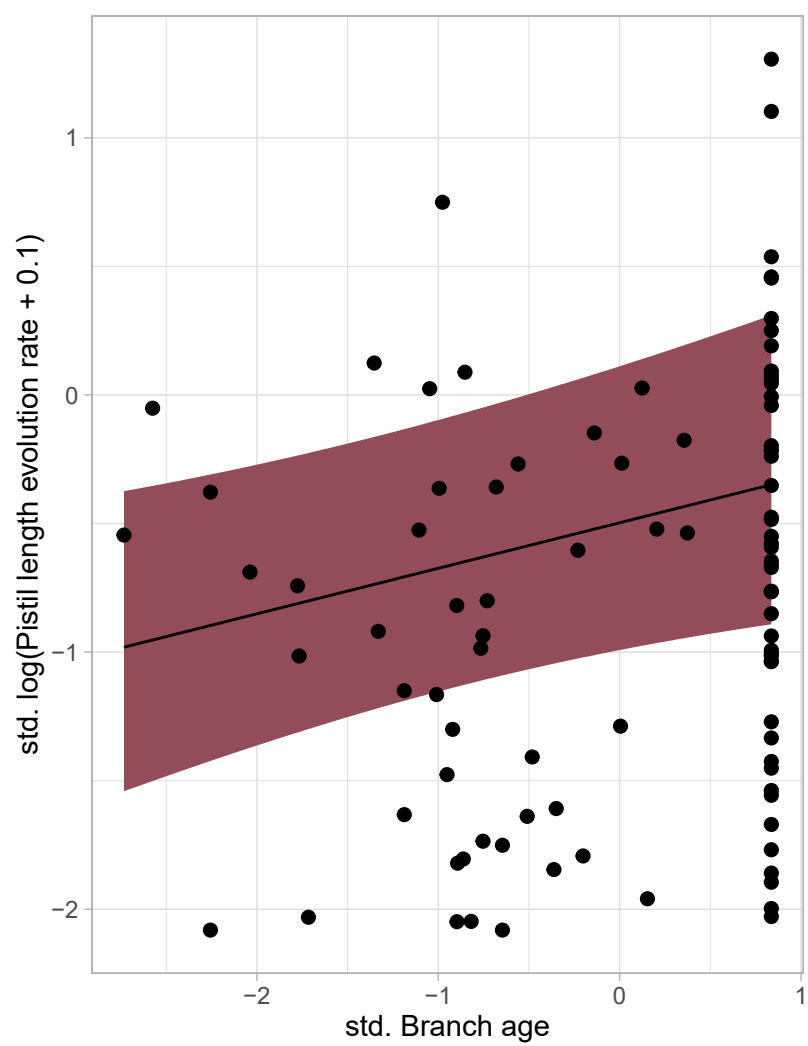

Supplement: Supplementary file 6 — Figure S5. The relationship between (a) the estimated per branch rates of pistil length evolution and the estimated per branch rates of diversification, (b) the estimated per branch rates of diversification and the age of each branch, and (c) the estimated per branch rates of pistil length evolution and the age of each branch. [file EVL3-5-277-s002.pdf]
